# CAG-targeted brain-permeable therapy tested in biallelic humanized polyQ mouse models

**DOI:** 10.1016/j.omtn.2025.102496

**Published:** 2025-02-22

**Authors:** Magdalena Surdyka, Żaneta Kalinowska-Pośka, Anna Niewiadomska-Cimicka, Ewelina Jesion, Agnieszka Fiszer, Elisabeth Singer-Mikosch, Lorraine Fievet, Lukasz Przybyl, Nicholas S. Caron, Michael R. Hayden, Huu Phuc Nguyen, Yvon Trottier, Maciej Figiel

**Affiliations:** 1 Institute of Bioorganic Chemistry, Polish Academy of Sciences, Poznań, Poland; 2 Institute of Genetics and Molecular and Cellular Biology (IGBMC), INSERM U1258, CNRS UMR7104, University of Strasbourg, Illkirch, France; 3 Department of Human Genetics, Ruhr University Bochum, Bochum, Germany; 4 Centre for Molecular Medicine and Therapeutics, BC Children’s Hospital Research Institute, Department of Medical Genetics, University of British Columbia, Vancouver, BC, Canada

**Keywords:** MT: Oligonucleotides: Therapies and Applications, short hairpin RNA, shRNA, CAG repeats targeting, Huntington disease, spinocerebellar ataxia type 3, SCA3, AAV-PHP.eB, blood-brain barrier, systemic delivery, gene therapy, neurodegenerative disease

## Abstract

In polyglutamine (polyQ) diseases, including Huntington disease (HD) and spinocerebellar ataxia type 3 (SCA3), targeting the mutant CAG tract in mRNA could be a therapeutic strategy for lowering pathogenic protein. We explored the viability of this therapeutic strategy *in vivo* at the level of the reagent design, toxicity, systemic delivery, brain regions transduction, silencing efficiency, and allele preference. We designed a series of CAG-directed short hairpin RNAs (shRNAs) based on a previous A2 reagent, allele selective *in vitro*. Humanized HD (Hu^128Q/21Q^) and SCA3 (Ki^150Q/21Q^) mice with mutant ∼100 CAGs and normal 21 CAGs alleles were used to simulate biallelic conditions occurring in patients. We administered AAV-PHP.eB shRNAs-encoding vectors into the blood as an equivalent of non-invasive CAG-directed brain-targeted therapy crossing the blood-brain barrier. We demonstrate that optimized CAG-targeted A4(P10) and A4(P10,11) shReagents can lower mutant huntingtin and ataxin-3 protein and its aggregates by targeting brain regions selectively and with diminished toxicity compared to other tested shRNAs. The important considerations of the approach are the silencing efficiency depending on the transduction region and careful dose adjustment. Moreover, the CAG approach could be suitable to target somatic expansion. Our work paves the way toward developing the therapy for polyQ diseases, potentially shortening drug development.

## Graphical abstract

**Figure:**
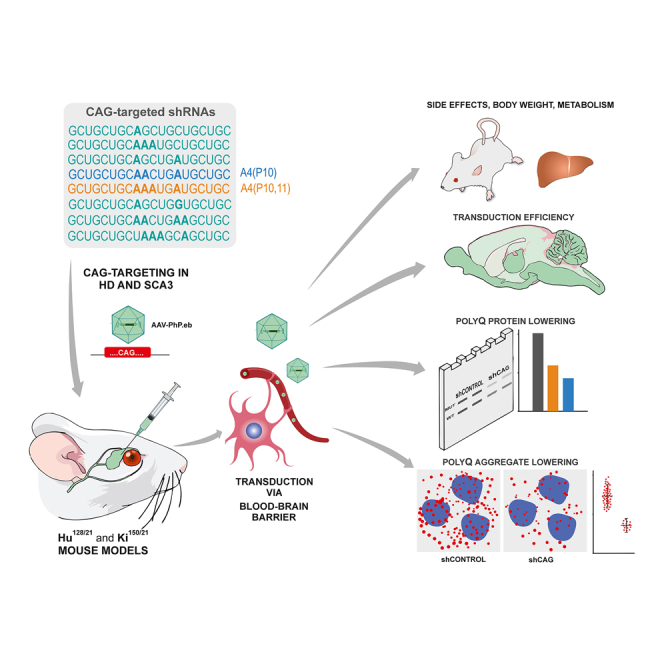

## Teaser

Polyglutamine (polyQ) disease preclinical therapy targeted the mutant RNA with CAG tract by short hairpin RNAs (shRNAs). shRNAs were delivered to the HD and SCA3 mouse brain via the blood-brain barrier, reducing pathogenic protein and aggregates with minimal toxicity. The approach could effectively treat polyQ diseases, potentially speeding up drug development.

## Introduction

Polyglutamine (polyQ) diseases are a group of the central nervous system (CNS) dominantly inherited disorders caused by the abnormal expansion of cytosine-adenine-guanine (CAG) repeats (usually >35–40 repeats) within the coding regions of the causative gene.^1^^,^^2^ The polyQ diseases, evoking brain neurodegeneration that results in progressive motor and often cognitive signs, comprise spinocerebellar ataxia (SCA1, -2, -3, -6, -7, and -17), Huntington disease (HD), and dentatorubral-pallidoluysian atrophy,^3^^,^^4^ among others.

The expanded CAG repeats are translated into toxic polyQ stretch, resulting in the deposition of misfolded protein in neurons, further impacting downstream phenotypes such as DNA damage, aggresome formation, dysregulation of mRNA, and protein production.^5^ The number of CAG repeats in causative genes often inversely correlates with an earlier age of onset.^6^ The somatic expansion phenomenon leads to further extension of the CAG tract and aggravation of the disease.^3^ The differences in CAG length between the two alleles of the affected gene, where the shorter allele still produces normal/wild-type (WT) protein, brought about the concept of therapeutic targeting of the long CAG tract. Importantly, allele-selective knockdown preserves the WT gene and protein function, which may have neuroprotective qualities.

CAG repeat tracts, which may undergo pathogenic expansion in HD and spinocerebellar ataxia type 3 (SCA3), are located in exon 1 of the *HTT* gene and exon 10 of the *ATXN3* gene.^7^^,^^8^ The CAG repeat-targeting approach is based on applying RNA molecules that may bind to multiple sites in the expanded repeat tract, relying on the microRNA (miRNA)-like or antisense mechanism. Therefore, mRNA with more CAG repeats may be silenced more effectively.^9^ Different CAG tract locations within the genes and CAG length may significantly affect the characteristics of CAG-targeted gene silencing.^10^

Blood-brain barrier (BBB)-permeable adeno-associated virus (AAV) vectors can deliver short hairpin RNA (shRNA) molecules for lowering mRNA and protein levels of disease-causative genes. The AAV vectors have specific cell types and brain region tropism^11^^,^^12^; therefore, they may be suitable as selective non-invasive therapeutic carriers for shRNA in polyQ diseases. In both diseases, the specific brain regions (cortex [CTX] and striatum [STR] in HD and cerebellum [CB] and brain stem [BS] in SCA3) are predominantly affected. Therefore, we drew attention to the efficiency of the potential therapy in different brain regions.^13^^,^^14^^,^^15^^,^^16^

This preclinical study aimed to develop and test a systemic, and therefore less-invasive CAG-targeted shRNA approach, for mutant polyQ protein reduction. The shRNA is delivered systemically across the BBB using the AAV-PHP.eB vector to directly target diseased brain cells in the CNS. To our knowledge, this is the first study to investigate the potential of system-delivered, vectorized CAG-targeted shReagents as a therapy in HD and SCA3 using humanized animal models that harbor human normal and mutant huntingtin protein (HTT) and similarly for human ataxin-3 protein (ATXN3). We used two humanized biallelic mouse HD and SCA3 models, Hu^128Q/21Q^ (HD)^17^ and Ki^150Q/21Q^ (SCA3),^18^ containing either human 128 or 150 CAG repeat mutant alleles and human normal 21 CAG alleles and containing no mouse endogens. Our primary A2 CAG-targeted reagent, previously shown as allele selective in cell culture, demonstrated *in vivo* toxicity when delivered as shRNA in AAV-PHP.eB.

Therefore, we aimed to diminish the adverse events by designing 8 new CAG-targeted shRNAs, testing their safety and efficacy in lowering HTT and ATXN3 protein level and evaluating their allele-selective potential. Our rationale with new reagents was to introduce multiple base substitutions into an almost pure CUG sequence of A2 to neutralize the toxicity and preserve the capacity to lower the level of mutant protein with a longer polyQ tract. As therapeutics for multiple polyQ diseases, our shReagents may accelerate drug development as they allow the use of common shRNAs for different diseases.

## Results

### Designing new shRNAs to target the CAG tract

Previous studies demonstrated that A2 or similar CAG-targeted reagents sequences were highly efficient and allele selective to lower polyQ protein levels *in vitro*.^19^^,^^20^^,^^21^^,^^22^ However, our *in silico* analysis for potential off-targets of A2 reagent showed full complementarity to several human genes (Table S1), disqualifying the reagent from further therapeutic development. This prompted us to design 8 new RNA sequences in which we made 1, 2, or 3 modifications relative to the A2 sequence (Table 1). Modifications are placed in the central or 3′ region of the reagent, which are locations different from the seed region crucial for miRNA activity. Incorporating more mismatches in the RNA sequence, as compared to A2, should still result in a reduced mutant polyQ protein level while not affecting the WT form. Subsequently, we tested the sequence of the designed shReagents for *in silico* off-targets and revealed minor potential off-targets for 8 shReagents selected for the present study (Table S1). We particularly noticed that previously used A4 and two of our shReagents—A4(P10A) or A4(P10,11A)—showed minor complementarity to mRNAs of genes that may be essential for neuronal physiology.

**Table 1:** The sequence of shRNA constructs targeting CAG tract

Name	Sense	Loop	Antisense
shScrambled	GGCCCAGCCGUAGCCGAGUG	AACUUCCUGUCAUU	CACUCGGCUACGGCUGGGCCUUUUU
A2	GCAGCAGCAGC**U**GCAGCAGC	UGCUUCCUGUCACA	GCUGCUGC**A**GCUGCUGCUGCUUUUU
A2(P10,11A)	GCAGCAGCA**UUU**GCAGCAGC	UGCUUCCUGUCACA	GCUGCUGC**AAA**UGCUGCUGCUUUUU
A4	GCAGC**U**GCAGC**U**GCAGCAGC	UGCUUCCUGUCACA	GCUGCUGC**A**GCUGC**A**GCUGCUUUUU
A4(P10A)	GCAGC**U**GCAG**UU**GCAGCAGC	UGCUUCCUGUCACA	GCUGCUGC**AA**CUGC**A**GCUGCUUUUU
A4(P10,11A)	GCAGC**U**GCA**UUU**GCAGCAGC	UGCUUCCUGUCACA	GCUGCUGC**AAA**UGC**A**GCUGCUUUUU
AG4	GCAGC**C**GCAGC**U**GCAGCAGC	UGCUUCCUGUCACA	GCUGCUGC**A**GCUGC**G**GCUGCUUUUU
AA4	GCAG**UU**GCAG**UU**GCAGCAGC	UGCUUCCUGUCACA	GCUGCUGC**AA**CUGC**AA**CUGCUUUUU
A15	GCAGC**U**GC**UUU**AGCAGCAGC	UGCUUCCUGUCACA	GCUGCUGCU**AAA**GC**A**GCUGCUUUUU

In antisense strands, the sequences designed to interact with CAG repeat tract in mutant genes are underlined, and nucleotides forming mismatches while interacting with the CAG tract are marked in bold.

### Distribution, transduction efficiency, HTT protein level, and tolerability 3 weeks post-injection of 8 new AAV-PHP.eB_EGFP_shRNAs in the HD mouse model

**Figure 1:**
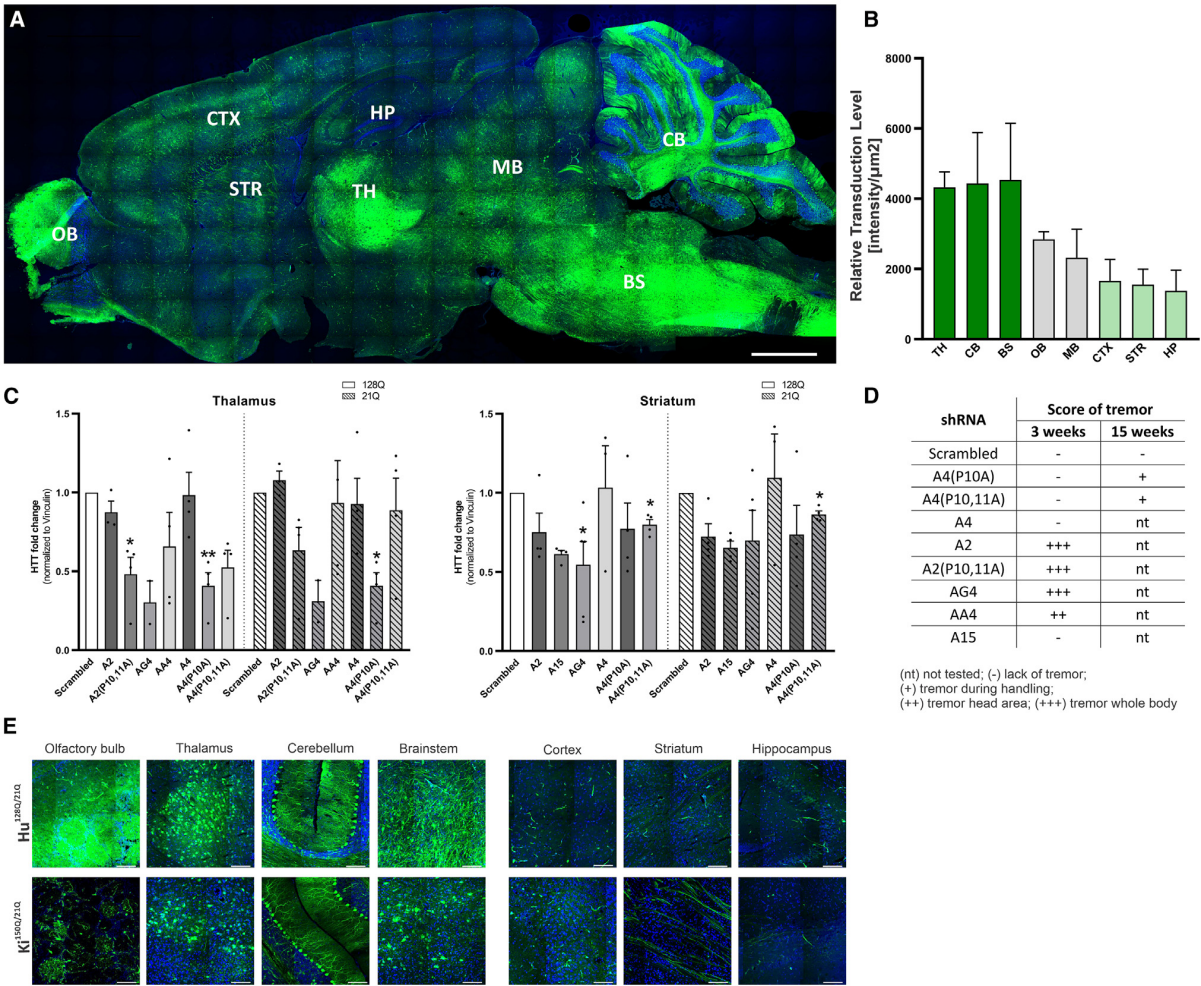
Distribution, transduction efficiency, side effects, and lowering HTT protein of AAV-PHP.eB_EGFP_shRNA in polyQ mouse models (A) Illustration of EGFP signal distribution in Hu^128Q/21Q^ brain structures performed 3 weeks post-retroorbital injection of AAV-PHP.eB_EGFP_shRNA (dose: 1.5 × 10^13^ vg/kg). Magnification 20×, scale bar, 100 μm. (B) The graph shows the relative transduction level as EGFP fluorescence intensity (RawIntDen) per square micrometer (intensity/μm^2^) in the 8 transduced brain structures 3 weeks post-injection. The dark-green bars show intensely transduced brain structures TH, CB, and BS, while light-green bars correspond to structures CTX, HP, and STR, showing weaker transduction. The data show mean ± the standard error of the mean (SEM) as error bars (*n* = 3). (C) Evaluation of efficiency in lowering HTT protein in the TH and STR 3 weeks post-injection of 8 shRNA shReagents (doses: 1.5 × 10^13^ and 0.5 × 10^13^ vg/kg for A2). (D) The table presents the tremor score in Hu^128Q/21Q^ mice at 3 weeks and in Hu^128Q/21Q^ and Ki^150Q/21Q^ mice at 15 weeks post-injection AAV-PHP.eB_EGFP_shRNA. In (C)the fold change was calculated from mean and presented ± SEM as error bars (*n* = 2, 3, or 4). The mean of shScrambled are set as value of 1 fold change vs shReagents. Means (respective shReagents and shScrambled) were analyzed with the Student’s t test; ∗*p* < 0.05; ∗∗*p* < 0.01. (E) Assessment of EGFP signals in brain structures of Hu^128Q/21Q^ (top) and Ki^150Q/21Q^ mice (bottom) 15 weeks post-injection. Magnification 40×; scale bar, 1000 μm. Brain regions: brain stem (BS), cerebellum (CB), cortex (CTX), hippocampus (HP), midbrain (MB), olfactory bulb (OB), and striatum (STR). Photographs show EGFP (green) and DAPI (blue).

We evaluated distribution and transduction efficiency in 8 brain structures of the Hu^128Q/21Q^ mouse model 3 weeks post-systemic delivery of new AAV-PHP.eB_EGFP_shRNAs (Figures 1A and S1). We measured the fluorescence intensity of EGFP signal in mouse brain regions indicated by regions of interest (ROIs) (Figure S2), 3 weeks post-injection, with viral vectors at a dose of 1.5 × 10^13^ vector genomes (vg)/kg for all shReagents except A2, at the dose 0.5 × 10^13^ vg/kg. EGFP signal was observed in the olfactory bulb (OB), CTX, STR, hippocampus (HP), thalamus (TH), midbrain (MB), CB, and BS (Figure 1A). The evaluation of relative transduction level (intensity/μm^2^) in individual brain structures revealed high EGFP signal intensity per μm^2^ in TH, CB, and BS, which was on average 2.9 fold greater than in CTX, HP, and STR, which demonstrated the weakest EGFP fluorescence per μm^2^. The OB and MB showed moderate transduction (Figure 1B). We investigated the transduction variability among cells using the Purkinje cell bodies in lobule 5 as a reference in 3 cerebella. We demonstrated that the transduction efficiency of each brain was similar at the level of single cells and presented a similar payload.

The potential to reduce the mutant HTT protein 3 weeks after delivery of shReagents was tested in 2 brain structures of HD mice: STR and TH (Figures 1C and S3). The well-transduced TH showed a statistically significant lowering of mutant HTT protein by 59% with A4(P10A) and by 52% with A2(P10,11A) compared to the shScrambled (*p* = 0.0085 and *p* = 0.0128, respectively). A tendency for HTT reduction was also observed after injection of A4(P10,11A) by 47% and AG4 by 70% compared to the shScrambled (*p* = 0.0986 and *p* = 0.1593, respectively) (Figures 1C and S1). In the STR, which represented a region with a relatively weak level of transduction, a statistically significant reduction in mutant HTT protein was observed after injections of AG4 by 45% (*p* = 0.0432) and A4(P10,11A) by 20% (*p* = 0.0432), while the A2 by 25%, A15 by 39%, and A4(P10A) by 23% demonstrated a tendency for reduction (*p* = 0.2258, *p* = 0.1059, and *p* = 0.2661, respectively) compared to shScrambled (Figures 1C and S1). Our study showed that the shReagents also lowered to some extent the normal HTT protein. In addition, we conducted observations of the behavioral phenotype over 3 weeks post-injection. During the first 10 days, we did not observe any adverse events or tolerability issues related to the injection of shReagents. On the days following, we observed increasing tremors in groups of mice injected with A2 and A2(P10,11A), AG4 (whole-body tremor), and AA4 group (head area) (Figure 1D). The mice injected with the other 4 shReagents (A4, A4(P10A), A4(P10,11A), A15) and shScrambled did not show adverse events until week 3 post-injection (Figure 1D). Due to the poor condition of the mice in the A2, A2(P10,11A), AG4, and AA4 groups, the entire experiment was terminated after 3 weeks. Based on the results of *in silico* analysis for potential off-targets and lowering HTT protein in short-term experiments (3 weeks), we chose 2 shReagents—A4(P10A) and A4(P10,11A)—for further long-term analyses (15 weeks).

### Distribution and tolerability 15 weeks post-injection of 2 selected AAV-PHP.eB_EGFP_shRNA in HD and SCA3 mouse models

We evaluated the transduction of brain regions of Hu^128Q/21Q^ and Ki^150Q/21Q^ mice 15 weeks post-injection of AAV-PHP.eB_EGFP containing shRNAs (Figure 1E). The representative images of 7 brain structures of Hu^128Q/21Q^ (upper panel) and Ki^150Q/21Q^ (lower panel) mice showed high transduction 15 weeks post-injection of the same regions as after 3 weeks (Figures 1A and 1E). Like before, and in both models, OB, TH, CB, and BS regions were prominently transduced, while CTX, STR, and HP demonstrated lower transduction (Figure 1E). This demonstrates that the EGFP signal was very stable up to 15 weeks after retroorbital injections (Figures 1A and 1E).

We assessed the tolerability in both Hu^128Q/21Q^ and Ki^150Q/21Q^ models after injections of A4(P10A) and A4(P10,11A) shReagents and found that individual animals showed a minor tremor or tremor during handling phenotype at 4 weeks. This phenotype was absent in the scramble-treated group (Figure 1D). We did not notice any further aggravation of symptoms and no other adverse events.

### A4(P10A) and A4(P10,11A) efficacy in lowering mutant polyQ protein in brain regions in HD and SCA3 mouse models 15 weeks post-injection

**Figure 2:**
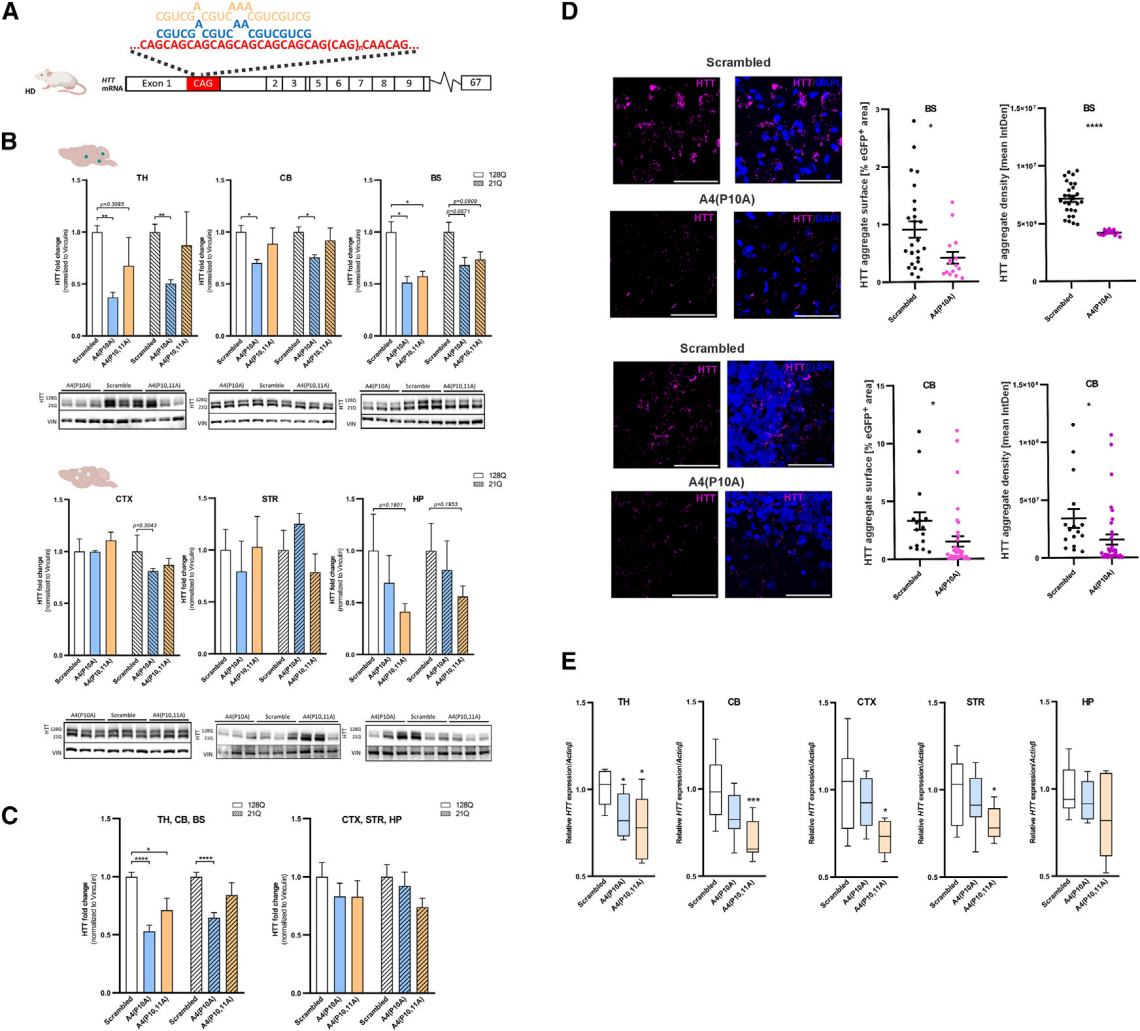
Lowering effect of shRNAs in brain regions of HD mouse model (A) Schematic of A4(P10A) (blue) and A4(P10,11A) (yellow) binding on the CAG tract in exon 1 of the *HTT* mRNA. (B) Quantification of HTT protein in the TH, CB, BS, CTX, STR, and HP of Hu^128Q/21Q^ mice injected with AAV-PHP.eB expressing A4(P10A) and A4(P10,11A) and shScrambled (1.5 × 10^13^ vg/kg). Brains with green dots in the corner show transduced brain regions; (top) well-transduced (TH, CB, and BS, dark green dots) and weakly transduced (CTX, STR, and HP, light green dots) brain structures. Vinculin was used as a control protein for normalization. Representative western blot images are shown under graphs; 128Q represents mutant HTT protein and 21Q represents WT HTT protein. (C) Collective lowering of HTT protein in well- (left) and weakly transduced (right) brain structures. (D) HTT aggregates were evaluated by immunohistochemical staining of the BS and CB with the anti-HTT EM48 antibody. The graphs shows data of HTT aggregate surface as a percentage of EGFP^+^ area (transduced cell area) and the data of HTT aggregate density (mean IntDen) in the BS or CB 15 weeks post-injection with A4(P10A) as shReagent and control shScrambled delivered in vector AAV-PHP.eB (*n* > 5 areas in brain structure in each mouse, *n* = 3). (E) Relative mRNA level of *HTT* (fold change) in various brain regions of the HD mouse model post-injection A4(P10A) and A4(P10,11A). Relative *HTT* transcript levels were measured by qPCR and normalized by the *Actinβ* gene expression level. The mRNA was extracted from the TH, CB, CTX, STR, and HP of Hu^128Q/21Q^. In (B-C) the fold change was calculated from mean and presented ± SEM as error bars (*n* = 3). In (B-C) the mean of shScrambled are set as value of 1 fold change vs shReagent on the graphs. The (B and C) means were analyzed with a Student’s t test. The data in (D) were analysed with Mann-Whitney test and ploted on graphs as median ±ranks. The data in (E) were analysed with Kruskal-Wallis test for multiple comparisons and subsequently post hoc analysed with Dunn test for pairwise comparison and the parameters were ploted as box plot. The p values in tests were indicated as follows ∗*p* < 0.05; ∗∗*p* < 0.01; ∗∗∗*p* < 0.001; ∗∗∗∗*p* < 0.0001.

**Figure 3:**
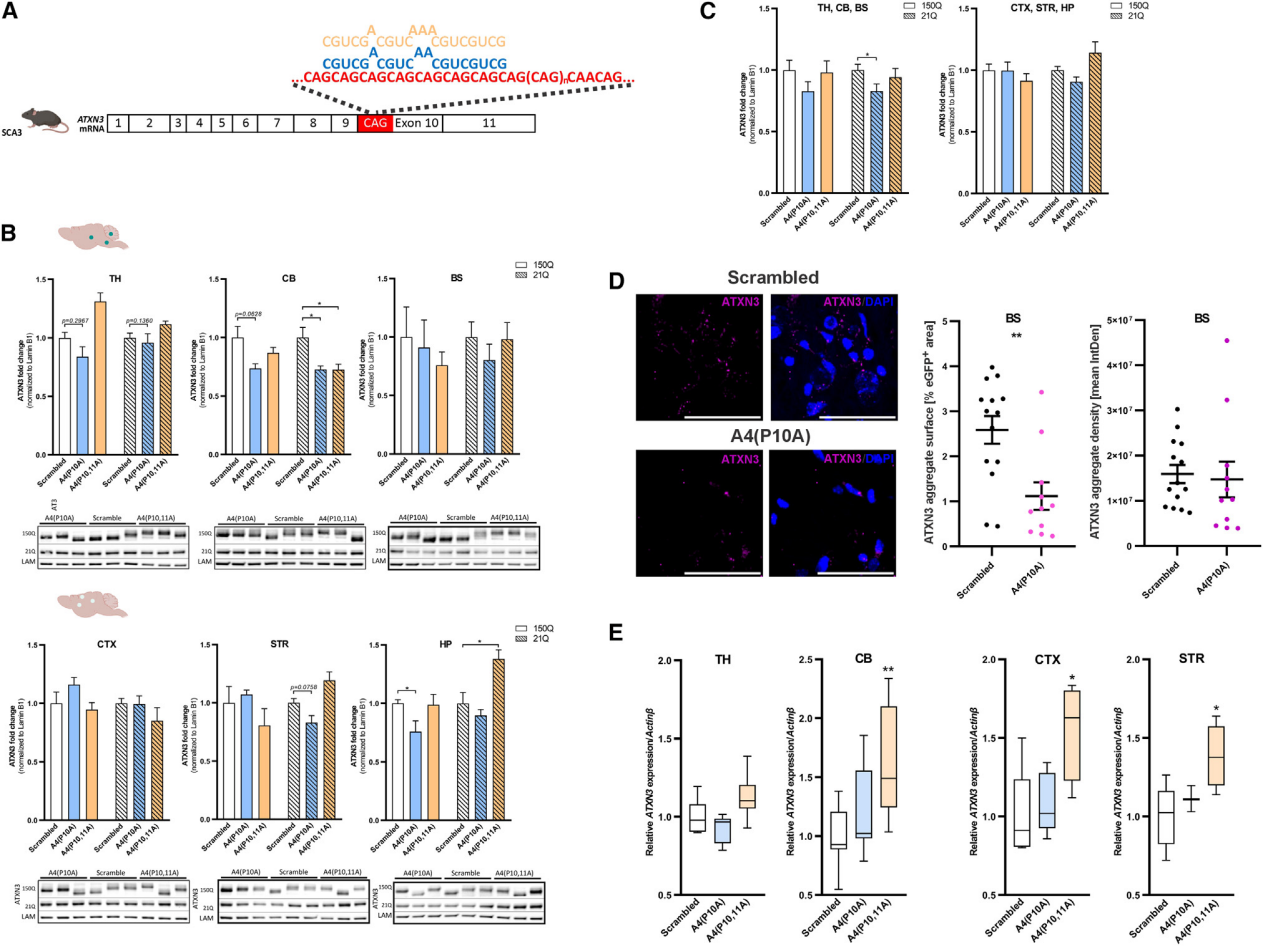
Lowering effect of shRNAs in brain regions of SCA3 mouse model (A) Schematic of A4(P10A) (blue) and A4(P10,11A) (yellow) binding on the CAG tract in exon 10 of the *ATXN3* mRNA. (B) Quantification of ATXN3 protein in the TH, CB, BS, CTX, STR, and HP of Ki^150Q/21Q^ mice injected with AAV-PHP.eB expressing A4(P10A) and A4(P10,11A) and shScrambled (1.5 × 10^13^ vg/kg). Brains with green dots in the corner show transduction in the brain region; (top) well-transduced (TH, CB, and BS, dark-green dots) and lower weakly transduced (CTX, STR, and HP, light-green dots) brain structures. Lamina B (LAM) was used as a control protein for normalization. Representative western blot images are shown under graphs; 21Q represents normal ATXN3 and 150Q represents mutant ATXN3 protein. The differences in PAGE mobility apparent on blot pictures are due to CAG expansion/contraction and the resulting differences in the length of the polyQ tract in the mutant ATXN3 protein in individual mice. (C) The collective lowering of ATXN3 protein in well (top) and weakly transduced (bottom) brain structures. (D) ATXN3 aggregates were evaluated by immunohistochemical staining of the BS with the anti-ATXN3 clone 1H9 antibody. The graph shows the data of ATXN3 aggregate surface expressed as a value of percentage of eGFP^+^ area (transfected cell area) and the data of ATXN3 aggregate density expressed as mean IntDen in the BS 15 weeks post-injection, with A4(P10A) as shReagent and control shScrambled delivered in vector AAV-PHP.eB (*n* > 5 area in brain structure in each mouse, *n* = 3). (E) Relative mRNA level of *ATXN3* (fold change) in various brain regions of the SCA3 mouse model post-injection A4(P10A) and A4(P10,11A). Relative *ATXN3* transcript levels were measured by qPCR and normalized by *Actinβ* gene expression level. The mRNA was extracted from the TH, CB, CTX, and STR Ki^150Q/21Q^. In (B and C) the fold change was calculated from mean and presented ± SEM as error bars (*n* = 3). The mean of shScrambled are set as value of 1 fold change vs shReagents on the graphs. The (B–C) means were analyzed with Student’s t test; The data in (D) were analysed with Mann-Whitney test and ploted on graphs as median ±ranks. The data in (E) were analysed with Kruskal-Wallis test for multiple comparisons and subsequently post hoc analysed with Dunn test for pairwise comparison and the parameters were ploted as box plot. The p values in tests were indicated as follows ∗*p* < 0.05; ∗∗*p* < 0.01; ∗∗∗*p* < 0.001; ∗∗∗∗*p* < 0.0001.

Schematics of A4(P10A) and A4(P10,11A) shRNA binding on the CAG tract in *HTT* exon 1 and *ATXN3* exon 10 mRNAs are shown in Figures 2A and 3A, respectively. We analyzed the effect of these bindings on the level of polyQ protein by western blotting in brain regions that showed relatively high and low levels of viral transduction (dark and light green dots, respectively in Figures 2B and 3B). Comparing the efficiency of shReagents in lowering mutant HTT protein in Hu^128Q/21Q^ mice, the most significant decreases were observed with A4(P10A) shReagent in all 3 well-transduced brain regions, with a 63% reduction (*p* = 0.0013) in the TH, 49% in the BS (*p* = 0.0137), and 30% in the CB (*p* = 0.0144) (Figure 2B, upper panels).

In contrast, there was no significant lowering effect with A4(P10A) in weakly transduced brain regions (CTX, STR, and HP) (Figure 2B, lower panels). We noted that A4(P10A) shReagent also lowered normal HTT in the well-transduced brain regions. The A4(P10,11A) only lowered mutant HTT protein in the BS by 43% (*p* = 0.0181) (Figure 2B). Interestingly, when combining the brain structures according to their transduction efficiency, both A4(P10A) and A4(P10,11A) showed significant lowering of the mutant HTT level in well-transduced regions (by 47%, *p* < 0.0001 and 29%, *p* = 0.0181, respectively) and no effect on weakly transduced regions (Figure 2C).

In contrast to HD mice, the lowering effects of A4(P10A) and A4(P10,11A) shReagents on mutant ATXN3 levels in Ki^150Q/21Q^ mice were only barely significant and showed no clear association with the transduction efficiency of brain structures (Figures 3B and 3C). The mutant ATXN3 level showed a tendency for reduction after A4(P10A) treatment vs. shScramble (by 24%, *p* = 0.0702) in the HP (Figure 3B). Interestingly, in the CB, a vulnerable structure in the pathology of SCA3, the mutant ATXN3 level was reduced by 26% (*p* = 0.0628) and the normal ATXN3 level by 27% vs. shScramble (*p* = 0.0411). The A4(P10,11A) shReagent reduced the level of normal ATXN3 by 27% *(p* = 0.0411) in the CB (Figure 3B). There was a tendency for reduction of mutant ATXN3 in combined well-transduced regions of the brain by 17% (*p* = 0.1437) with A4(P10A) (Figure 3C).

In both HD and SCA3 mouse models, A4(P10A) and A4(P10,11A) shReagents did not demonstrate a clear allele selectivity toward lowering mutant polyQ proteins (Figures 2C and 3C).

### A4(P10A) and A4(P10,11A) efficacy in lowering aggregates in brain regions in HD and SCA3 mouse models 15 weeks post-injection

Hu^128Q/21Q^ and Ki^150Q/150Q^ mouse models show protein aggregates in several brain regions.^17^^,^^18^^,^^23^ We performed immunostaining using EM48 anti-HTT antibody to determine the effect of the CAG-targeted shReagents on aggregate formation in the CB and BS of HD mice (Figure 2D). We determined the percentage of the area of aggregates and the mean integrated density of aggregates 15 weeks post-injection with A4(P10A) vs. shScrambled as the control. We observed a decrease in the percentage of the area of aggregates in the HD BS and CB by 58% and 55% (*p* = 0.0142 and *p* = 0.0374), respectively (Figure 2D) and a significant reduction by 59% and 20% in the mean integrated density of aggregates in the Hu^128Q/21Q^ mice in the same brain structures (*p* < 0.0001 and *p* = 0.0347, respectively) (Figure 2D). In the SCA3 model, the percentage of the area of aggregates detected by anti-ATXN3 1H9 antibody in the area of the BS was decreased by 57% (*p* = 0.0031) (Figure 3D). The mean integrated density of aggregates in SCA3 mice showed no changes in the same group injected with A4(P10A) shReagent group vs. control shScrambled group in the BS.

### A4(P10A) and A4(P10,11A) efficacy in lowering *HTT* and *ATXN3* mRNA in brain regions in HD and SCA3 mouse models 15 weeks post-injection

We measured statistically significant reduction of *HTT* transcript in the TH by 22% (*p* = 0.0220), CB by 29% (*p* = 0.0009), CTX by 27% (*p* = 0.0122), and STR by 21% (*p* = 0.0221) (Figure 2E) following injection with A4(P10,11A), whereas *HTT* transcript downregulation by 15% was only observed in the TH with A4(P10A) (*p* = 0.0311).

In the SCA3 mouse model, we observed no significant decrease in the *ATXN3* mRNA in selected brain structures after injecting the 2 shReagents. However, A4(P10,11A) shReagent significantly increased *ATXN3* mRNA levels in the CB, the CTX, and the STR (Figure 3E).

### Phenotypic mice changes in HD and SCA3 mouse models 15 weeks post-injection A4(P10A) and A4(P10,11A)

The characteristic of HD and SCA3 disease mouse models is the 30% weight gain of Hu^128Q/21Q^ mice over 3–4 months,^17^ while there is a stoppage in gaining weight in the Ki^150Q/21Q^ model compared to WT mice.^18^^,^^23^ Before injections, we determined the baseline phenotypic traits in Hu^128Q/21Q^ and Ki^150Q/21Q^ mice. On day 1, before injection, the phenotype scoring test (PST) revealed a mean score <1 baseline phenotype in all experimental groups. Before concluding the experiments, we repeated the PST.

**Figure 4:**
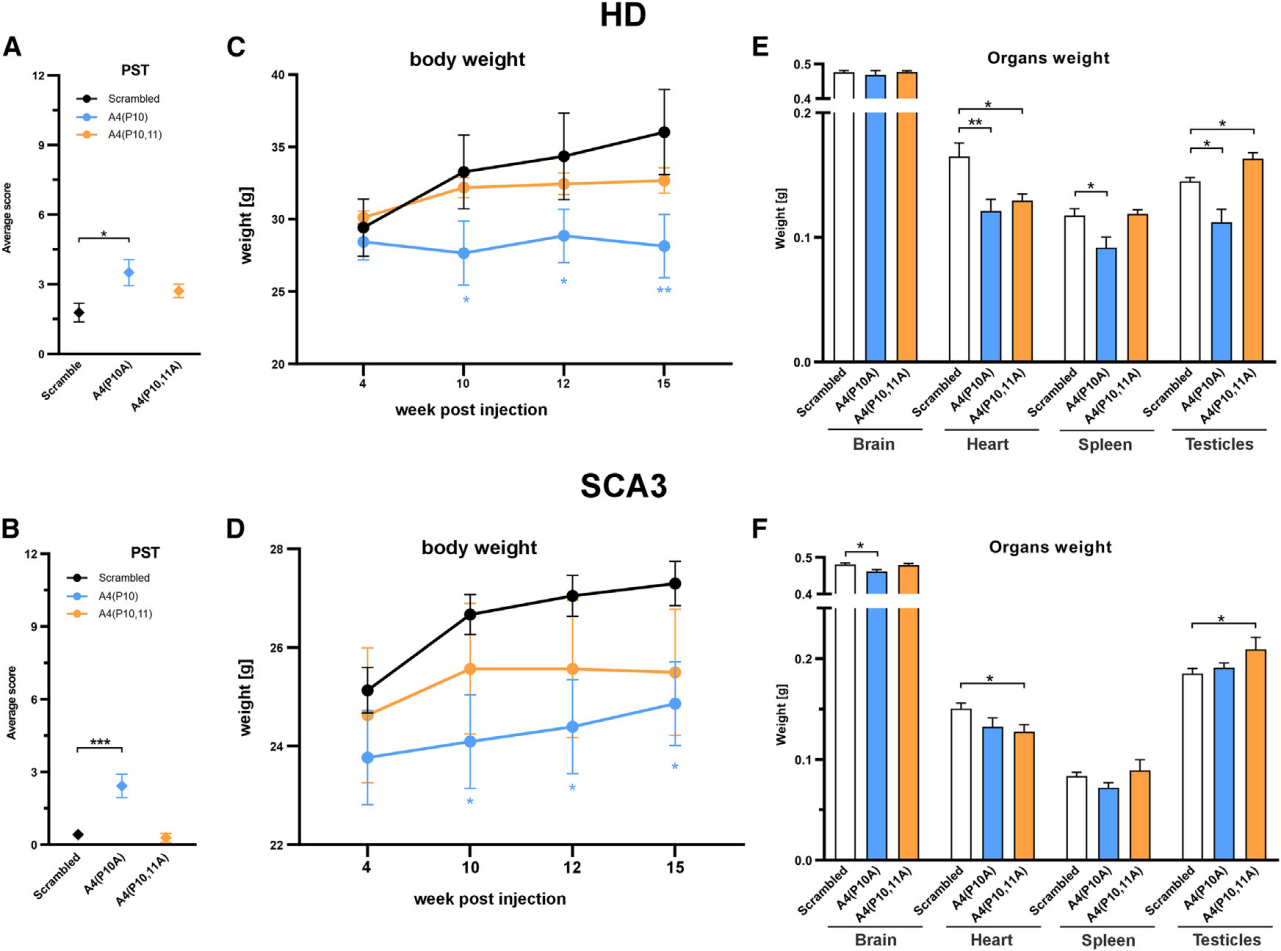
Phenotypic changes in HD and SCA3 mouse models 15 weeks post-injection AAV_PHP.eB_shRNA (A and B) The graph demonstrates the phenotype scoring test (PST) results, where 0 points are the normal condition and 12 points are the most affected mouse condition. The points were scored by animals 15 weeks post-injection and are expressed as average scores. (C and D) The graphs demonstrate differences in body weight gain of (C) HD and (D) SCA3 mice measured at 4, 10, 12, and 15 weeks post-injection with A4(P10A) (*n* = 8) and A4(P10,11A) (*n* = 9) and shScrambled (*n* = 11) shRNAs; the weight is expressed in grams. (E and F) Postmortem organ weights of brain, heart, spleen, and testis were recorded 15 weeks post-injection. The PST and organ weight data represent the mean ± SEM as error bars. Means (shReagent and shScrambled) were analyzed with Student’s t test; ∗*p* < 0.05 (*n* = 6). The body weight data were analyzed by two-way ANOVA (*p* < 0.05) and Fisher’s least significant difference post hoc (∗*p* < 0.05; ∗∗*p* < 0.01).

After 15 weeks, we observed a slight deterioration in the phenotype (increased score) in some animals with A4(P10A) and A4(P10,11A); however, the score never exceeded 4.5 mean average score points out of the 12-point scale in any of the experimental groups. We observed the highest point score upon A4(P10A) injection in Hu^128Q/21Q^ and Ki^150Q/21Q^ mice (*p* = 0.0233; *p* = 0.0001, respectively) compared to shScrambled (Figures 4A and 4B). The body weight in both HD and SCA3 animals was assessed in the shReagent groups vs. the shScrambled-treated group at 4, 10, 12, and 15 weeks post-injection. The HD animals injected with A4(P10A) demonstrated decreased body weight by 9.2% (*p =* 0.0389), 9.9% (*p =* 0.0430), and 10.1% (*p* = 0.0041) at 10, 12, and 15 weeks post-injection, respectively. The results demonstrate that HD animals injected with A4(P10) ceased to gain weight compared to shScrambled (Figure 4C). Also, the SCA3 animals demonstrated decreased body weight by 6.4% (*p* = 0.0367), 6.6% (*p* = 0.0329), and 6.8% (*p* = 0.031) at 10, 12, and 15 weeks post-injection, respectively (Figure 4D). There was no significant change in body weight in HD and SCA3 mice injected with A4(P10,11A) (Figures 4C and 4D).

In addition, at week 15, we analyzed the weights of the postmortem brain, heart, spleen, and testes organs (Figures 4E and 4F). In Hu^128Q/21Q^ mice, we observed a statistically significant reduction in heart and testis weight after injection of A4(P10A) and A4(P10,11A) (*p* = 0.0087 and *p* = 0.0131, respectively, for heart; *p* = 0.0386 for testis) (Figure 4E). For A4(P10A) shReagent, a statistically significant weight reduction was also observed in spleen weight in the HD mouse model (Figure 4E). In the SCA3 model, after the A4(P10A) shReagent injection, we observed a slight decrease in brain weight by 4% (*p* = 0.0164) (Figure 4F). A4(P10,11A) led to a reduction in heart weight by 15% (*p* = 0.0336) and an increase in testis weight by 13% (*p* = 0.0435) (Figure 4F).

It was remarkable to note that while Hu^128Q/21Q^ mice had a 27% higher body weight than Ki^150Q/21Q^ mice, the 2 models had similar brain weights (Figures 4E and 4F). However, the viral vector dose was calculated based on body weight (vg/kg). Assuming that brain uptake was equally effective in both models, the HD mouse brain received a higher dose than did the SCA3 mouse brain. This observation should be considered when designing future therapies and *in vivo* testing using genetic carriers such as AAVs with high tropism toward the brain.

### Evaluation of the impact of AAV-PHP.eB_EGFP_shRNA treatment on peripheral tissue 15 weeks post-injection

**Figure 5:**
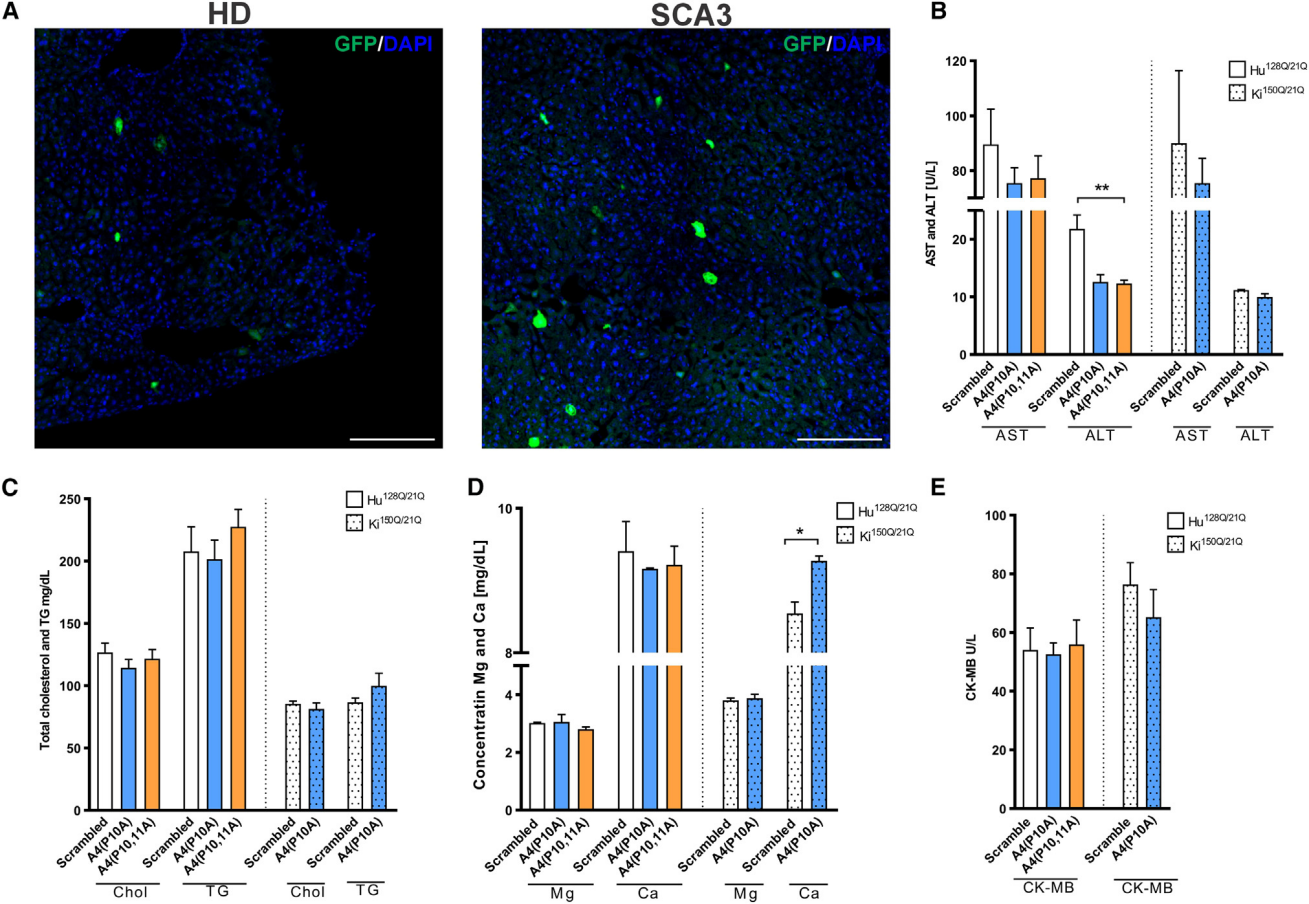
Liver transduction and biochemical parameters evaluation in HD and SCA3 mouse model The EGFP signal in representative liver sections of (A) Hu^128Q/21Q^ and Ki^150Q/21Q^ mice. (B–E) (B) The change in AST and ALT parameters, (C) level of total cholesterol (Chol) and triglycerides (TGs), (D) concentrations of magnesium (Mg) and calcium (Ca), and (E) creatine kinase (CK-MB) in the serum of Hu^128Q/21Q^ and Ki^150Q/21Q^ mice 15 weeks after AAV-PHP.eB_shRNA injections. The data shown in (B)–(E) are mean ± SEM as error bars (*n* = 3 or 4), and the analysis compares shReagent- vs shScrambled -treated mice using Student’s t-test; ∗*p* < 0.05; ∗∗*p* < 0.01.

Future safe therapy using BBB-permeable vectors aims to reach the brain and transduce brain cells selectively. However, AAV vectors may transduce peripheral tissues, such as the transduction of the liver previously demonstrated for several serotypes.^24^ To determine whether the liver is transduced in our systemic delivery of CAG-targeted shReagents, we examined EGFP signal in liver sections collected from experimental animal groups. In liver sections from both polyQ mouse models, the EGFP transduction signal was present in less than 1.5% of all cells (Figure 5A).

Upon blood collection at 15 weeks, we monitored alanine aminotransferase (ALT) and aspartate aminotransferase (AST) levels, critical indicators of liver function. The levels of these enzymes showed just minor differences in mice injected with A4(P10A) or A4(P10,11A) vs. shScrambled-treated groups in both mouse models. The ALT was significantly decreased after A4(P10A) and A4(P10,11A) (Figure 5B), which may indicate a reduction in liver inflammation in Hu^128Q/21Q^. We conclude that systemic injections of AAV-PHP.eB have a minor effect on liver transduction and do not negatively impact liver biochemical parameters.

We also evaluated selected biochemical parameters associated with changes in lipid metabolism: cholesterol (Chol) and triglyceride (TG) levels (Figure 5C), the concentration of magnesium and calcium (Mg, Ca) (Figure 5D), and the cardiac-specific subform of creatine kinase (CK-MB) (Figure 5E). Although the basal Chol and TG levels differed between the HD and SCA3 models, we did not observe statistically significant changes between treatment with shReagents vs. control shScrambled (Figure 5C). Measurements of Mg and Ca levels demonstrated a minor increase in Ca levels after A4(P10A) shReagent injection into Ki^150Q/21Q^ (*p* = 0.0132) (Figure 5D). Evaluation of another essential parameter, CK-MB, showed no changes after the administration of shReagents in AAV-PHP.eB (Figure 5E). The lack of adverse changes in liver parameters and other critical biochemical parameters allows us to assume that the applied therapy did not harm the overall body condition of mice, which is very promising for implementing CAG-targeting brain-permeable gene therapies using viral vectors.

## Discussion

CAG trinucleotide repeat expansions translating into an abnormally long polyQ stretch in encoded toxic proteins are the cause of neurodegenerative polyQ diseases. Therefore, lowering toxic protein levels by antisense oligonucleotides or RNA interference (RNAi) is a promising therapeutic approach.^14^^,^^20^^,^^25^ The application of these molecules lowers the pathogenic protein level through RNase H or Ago2-mediated RNA cleavage. An alternative approach for CAG targeting would be designing small interfering RNAs (siRNAs) that mimic miRNA activity to reduce protein levels efficiently by translational inhibition.^5^^,^^10^

Inducing the toxic protein lowering by siRNA can be limited by ineffective drug delivery to the CNS and diseased cell populations, even when direct brain injections are used. Moreover, direct delivery is invasive and may cause medical complications. Therefore, we tested our CAG-targeted shReagents in retroorbital systemic delivery of the AAV-PHP.eb serotype vector.^26^ Such blood administration can be considered non-invasive compared to direct brain or spinal cord injection in patients. The serotype can cross the BBB and bind to populations of neurons and astrocytes potentially affected in the pathogenesis. *HTT* is expressed at high levels across the brain (https://www.proteinatlas.org/ENSG00000197386-HTT/brain)^27^; therefore, the AAV-PHP.eB serotype may be very attractive as a therapeutic tool in HD.

In our therapeutic approach, in both Hu^128Q/21Q^ and Ki^150Q/21Q^ models, we observed robust transductions in the OB, TH, CB, and BS by AAV-PHP.eB; the STR, CTX, and HP also were transduced but with lower efficiency (Figures 1A, 1B, and 1E). The diversified levels of transduction among brain regions cannot be attributed to the differences in potency or selectivity of cytomegalovirus (CMV) promoters or the stability of EGFP. This is because the modern recombinant and enhancer-containing hybrid CMV promoters induce broad tissue expression and are potent and silencing-resistant recombinant versions patented as early as 2011.^28^ Therefore, these efficient new-generation CMV tools, featuring resistance to silencing in stem cells and a wide variety of cells, are used to generate transgenic mice and for AAV-based therapies, such as Glybera (the first approved gene therapy), Luxturna, and Zolgensma. In addition, systemic injection of AAV vectors could lead to a fluctuation as demonstrated previously by Mathiesen et al. and Smith et al.,^29^^,^^30^ who showed 12%–50% and 5%–15% transduced cells, respectively. In our case, we observed that this fluctuation in transduction levels was more important in highly transduced regions such as OB and TH, than low-transduced regions (CTX, HP, STR, and MB) (Figure 1B).

The transduction and the EGFP signal were stable over time, after 3 weeks (Figure 1A) and 15 weeks (Figure 1E) post-injection. Lastly, the differential transduction of brain regions and EGFP signal intensity had similar patterns in both humanized Hu^128Q/21Q^ and Ki^150Q/21Q^ biallelic models despite their different genetic backgrounds (FVB and C57). The systemic delivery of AAV-PHP.eB containing shRNA coding sequence was also safe for peripheral tissues in Hu^128Q/21Q^ and Ki^150Q/21Q^ animals since we only discovered single cells transduced in the liver (<1.5%) and minor changes in biochemical blood parameters in injected animals (Figure 5).

The safe use of gene therapies requires the evaluation of potential off-targets for the tested RNA sequence. Our *in silico* analyses demonstrated potential off-targets for A2 (Table S1), our first-generation CAG-targeted molecule. Despite delivering 3 times lower doses of A2 than other shRNAs, adverse effects of the A2 were detected by short-term behavioral observations showing whole-body tremor symptoms (Figure 1D). Therefore, we designed new shReagents around the concept of decreasing the direct homology of the shReagents to CAG repeat sequences to fulfill 2 aims. The first aim was to diminish the original toxicity of A2 shReagent. Indeed, we found that increasing the degree of A and G substitutions in the shReagent sequence resulted in the lowering of the number of *in silico* off-target genes, indicating a potential lowering in toxicity. The second aim was that despite the lower complementarity of such shReagents to the CAG tract, we expected to preserve significant silencing efficiency. We hoped that the availability of multiple target sequences in long CAG would result in sufficient synergistic silencing by miRNA-like or another mechanism.^19^^,^^20^^,^^21^^,^^22^ The shReagents series derived from A2 containing addition substitutions such as A2(P10A) and A2(P10,11A) showed slightly less *in silico* off-targets than A2 but more off-targets than shReagents of the A4 series, A4(P10A) and A4(P10,11A). In line with the frequency of genetic off-targets identified *in silico*, our initial screening demonstrated that the molecules with fewer off-targets derived from A4 (Figure 1D; Table S1) had substantially fewer adverse events, even in high doses of the shReagent in AAV. In the case of the shReagents we dropped from the further analysis, their major adverse events may not necessarily arise from off-targeting. The pathogenic driver can be an excess of the antisense mRNA, particularly in the mouse brains, due to shRNA expression.^31^

Based on the minor adverse events during the initial selection and the efficiency of the mutant HTT reduction, we evaluated the lowering effect of the two shReagents, A4(P10A) and A4(P10,11A), in several brain regions in two polyQ mouse models in a long-term experiment 15 weeks post-administration of the shReagent. Our PST of the HD and SCA3 mice showed minor behavioral abnormalities 15 weeks after delivery of shReagents. Hu^128Q/21Q^ and Ki^150Q/21Q^ mice received no more than 4.5 points out of the total PST scale, where 12 points are the worst mouse condition. We did not observe the diminished health status of shReagent vs. shScrambled animal groups measured by indicative biochemical parameters at experiment termination. Although the ALT significantly decreased with A4(P10), it remained within the healthy normal level. In addition, we did not observe dramatic changes in the weight of organs in mice treated with the shReagents vs. shScrambled. There were moderate but statistically significant decreases in organ weight, occurring mainly in the HD model treated with the A4(P10). The effect may be attributed to decreased overall body weight and diminished organ fat content, indicating favorable therapeutic effects (Figures 4E, 4F, and 5B–5E).

Interestingly, the weight gain, increased fat accumulation, or even obesity seen in mice are the metabolic consequences of pathology in HD patients and models.^32^^,^^33^ Even localized overexpression of the mutant HTT in the hypothalamus causes increased weight gain observed in HD mice R6/2 and BACHD. At the late stages in R6/2, there is a weight decline; even then, fat accumulation itself remains increased.^34^

We evaluated body weight in shReagent-treated HD and SCA3 mice, demonstrating a diminished weight in animals injected with the A4(P10A) and A4(P10,11A) (Figures 4C and 4D). Similar weight lowering was reported in other therapeutic works, including the uniQure AAV5-shRNA,^35^ as a positive result of the therapeutic approach.

At 15 weeks after shRNA application, we evaluated the level of mRNA and protein for HTT and ATXN3 in HD and SCA3 mice, respectively, in several brain structures (Figures 2B–2E and 3B–3E). In the case of the HTT, the A4(P10A) and A4(P10,11A) shReagents prominently lowered the protein in brain regions where the transduction was relatively high (Figure 2B). The brain regions transduced to a lesser extent did not show protein lowering (Figure 2C). Only the HP showed a decrease in HTT protein. In the case of ATXN3 protein, we obtained a lowering in the CB, which is most critical in SCA3 pathogenesis (Figure 3B). In the case of aggregates after injection of A4(P10A), we observed a lowering of the surface and density of the HTT aggregates (Figure 2D). In the case of ATXN3 protein, we observed a lowering of the surface but not density of the aggregates (Figure 3D). Aggregates are a central hallmark of the disease. Therefore, their lowering in both models indicates promising therapeutic potential in polyQ diseases.

We have demonstrated the lowering of mutant protein levels by therapeutic shReagents that target CAG sequence in unique *in vivo* systemic delivery, which is a preferred approach in future therapies. We were able to lower the polyQ proteins in multiple brain regions, eradicating the mutant protein more broadly. We further show that future AAV therapies administered systemically should consider a precise level of brain tissue transduction. The level of transduction of a brain region may be significant but insufficient for protein lowering, which may also depend on the types of cells containing polyQ protein (cell populations transduced). Additionally, checking the effects of the therapy at the mRNA level in the case of *HTT*, the A4(P10,11A) lowered the mRNA level significantly in most of the brain structures tested, while no statistical lowering was noted for A4(P10A); however, some reduction was observed (Figure 2E). The effect of both shReagents on the *ATXN3* mRNA level was not uniform among brain structures and between injected animals (Figure 3E).

As demonstrated, targeting the CAG tract with shReagents in *in vivo* biallelic setup differs from *in vitro* conditions where reagent concentration, cell proliferation stage, and others can be precisely adjusted. Our work demonstrates that targeting mRNA containing a relatively short CAG tract (21 CAGs) may also lead to some lowering of the normal protein *in vivo*. Although we did not obtain high allele selectivity in this approach, the effective reduction of mutant HTT protein can be a therapeutic approach to different lengths of the CAG tract. Large somatic expansions in CAG tracts may worsen the disease progression.^36^^,^^37^^,^^38^^,^^39^

Therefore, an optimized therapeutic approach using our shReagents may be perfectly tailored to target very long repeats in pathogenic mRNA. Our shReagents are also highly active in lowering aggregates of mutant proteins with very long polyQ expanded tracts also recently shown by us in SCA7.^40^ The shReagents reduce the normal protein level only to some degree, which may preserve sufficient HTT for essential normal functions, which may not be the case when using classical siRNA. Therefore, the exact discrimination by CAG shReagent between alleles in length, like in our mice, could be a minor issue. In contrast, the discrimination between long, somatically expanded alleles could be a central consideration for the therapy using the shReagents.

The previous studies demonstrating allele selective lowering of mutant HTT were conducted on mouse models containing a long polyQ tract in human HTT and mouse HTT with only 5 CAG repeats. The setup is a method of choice since 5 CAG in such mouse *HTT* mRNA may be too short to test any preferential lowering of human mutant *HTT* by CAG-targeted reagents.^41^^,^^42^ Therefore, exposure of 21 CAG repeat tract in human *HTT* mRNA to CAG-targeted shReagents is expected to cause a more effective reduction of the corresponding 21Q protein. In the case of mutant ATXN3, the reagents targeting CAG previously^43^ reduced the protein and showed some degree of allele selectivity in precisely optimized *in vitro* cell culture conditions. However, even those optimized conditions were less effective for mutant ATXN3 than for lowering the HTT protein.^43^ The difference in lowering efficacy for HTT and ATXN3 protein may depend on the location of the CAG tract in the exons of corresponding genes.^10^

Our proposed approach indicates that CAG-targeted shReagents delivered in the AAV-PHP.eB viral vector across the BBB constitute a promising therapeutic approach in polyQ diseases since it is much less invasive, safe, does not affect peripheral tissues, and results are particularly promising for HD. Their potential for silencing of somatically expanded alleles should be investigated.

## Materials and methods

### Animals

The HD (Hu^128Q/21Q^) mouse model on the FVB genetic background was described earlier by Southwell et al.^17^ The Hu^128Q/21Q^ mouse model was created by crossing *Htt*^*−/−*^ Bac^21Q^ mice and *Htt*^*−/−*^ Yac^128Q^ mice on the *Htt*^−/−^ background. The SCA3 (Ki^150Q^) disease model was previously designed and described by Piasecki et al., Wiatr et al., and Switonski et al.^18^^,^^23^^,^^44^ The Ki^150Q/21Q^ model on a C57Bl/6J genetic background was created by crossing Ki^150Q/150Q^ (mut/mut) and Ki^21Q/21Q^ (mut/mut) and genotyped. The Hu^128Q/21Q^ model is humanized and contains full mutant *HTT* (128Q) and normal *HTT* (21Q) genes. The Ki^150Q/21Q^ biallelic humanized model contained mutant *ATXN3* (150Q) and normal *ATXN3* (21Q). The mice were bred and maintained in the animal facility of the Centre for Advanced Technologies of the Adam Mickiewicz University in Poznan, Poland. Animals were housed in individually ventilated cages on a 12-h light/dark cycle with water and food *ad libitum*. The experiments were approved by the Local Ethics Committee for Animal Experiments in Poznan (contract no. 64/2018). Animal stress levels were minimized during all procedures and animal handling. The intravenous administration of AAV vectors was performed by retroorbital injection of adult mice (mixed sex; 8–12 weeks of age, *n* = 4 for 9 shRNAs per experiment of 3 weeks and *n* = 6 for 3 shRNAs per experiment of 15 weeks) at a dose of 1.5 × 10^13^ vg/kg for all shRNAs (except for A2 0.5 × 10^13^ vg/kg; Table S2). The mice were observed, and their body weight was monitored over 15 weeks of experiments.

Whole blood was collected from the ventricle. Then, mice were transcardially perfused with ice-cold PBS and sacrificed 3 or 15 weeks after injection. Mice were anesthetized with isoflurane during injection, cardiac blood collection, and terminal perfusion. The blood was centrifuged to separate the plasma, and the tissues were collected and weighed. The plasma was analyzed for total Chol, TGs, AST), ALT, Mg, Ca, and CK-MB at the Biochemistry and Hematology Laboratory at the Centre for Advanced Technologies at Adam Mickiewicz University. The brains were dissected into structures, obtaining CTX, STR, HP, TH, CB, and BS.

### Phenotype scoring

The tremor score was assessed by observing the mice weekly for 3 or 15 weeks using the same experimental conditions. The following scale was used: (−) lack of tremor, (+) tremor observed during handling, (++) tremor of the head area, and (+++) tremor of the whole body. In the case of the acute and progressive worsening of the mouse condition, the experiment was terminated.

PSTs, designed to assess polyQ disease-related phenotype effects,^45^ were performed before injection and on the sacrifice day (15 weeks post-injections). The PST comprised four analyses: edge test, hindlimb clasping, gait, and kyphosis. Each of the four individual tests contributed to the overall test score, ranging from 0 to 12, representing the total phenotypic assessment. Individually, each test was scored on a scale of 0–3 (0 = no phenotypic symptoms, 1 = light phenotypic symptoms, 2 = medium phenotypic symptoms, 3 = prominent phenotypic symptoms).

### AAV plasmid and vectors

The AAV-PHP.eB particles were generated and purified at the Molecular Biology and Virus Service in the Institute of Genetics and Molecular and Cellular Biology in Illkirch, France. Titer measurements were conducted using qPCR during production, and vectors were diluted in sterile saline to achieve the final injection dosage (Table S2.). The viral constructs for all AAV-PHP.eB_EGFP_shRNA molecules contained EGFP reporter gene with a CMV promoter and the shRNA expression cassette, including H1 promoter controlling the expression of shRNA, which comprised a 22-bp stem and a 10-nt miR-23 loop (Table 1). The cassettes were flanked by AAV inverted terminal repeat sequences.

### RNA isolation and quantitative reverse-transcription PCR

Total RNA from mice brain fragments was isolated using TRIzol reagent (MRC) and phenol:chloroform:isoamyl alcohol (25:24:1). RNA quality and concentration were measured by UV spectrophotometry on NanoDrop.

Total RNA, 500 ng, was reverse transcribed into cDNA using MultiScribe (Thermo Scientific) according to standard protocol. The sequence of primers is listed in Table S3. Quantitative reverse-transcription PCR was performed on the CFX Connect real-time PCR detection system (Bio-Rad) using the EvaGreen Supermix (Bio-Rad) with *Actinβ* as the reference gene. The reactions were performed under specific thermal cycle conditions: denaturation at 95°C for 30 s, followed by 40 cycles of denaturation at 95°C for 15 s, and annealing at 60°C for 30 s. Gene expression levels were normalized to those of shScrambled-treated mice.

### Western blotting

Total protein lysates of mouse brain tissue were obtained by using lysis buffer (60 mM TRIS-base/2% SDS/10% sucrose) supplemented with a protease inhibitor cocktail (Roche), and sonication by Sonopuls mini 20 sonicator (Bandelin). The protein concentration of the obtained lysates was measured with the Pierce BCA Protein Assay Kit according to the manufacturer’s instructions using the Infinite 200 PRO NanoQuant Plate (Tecan) at 562 nm. Total protein, 20 μg, was separated on NuPAGE Tris-Acetate 3%–8% Protein Gel (Thermo Fisher Scientific) for HTT protein analysis and on 4%–15% Mini-PROTEAN TGXTM Precast Protein Gels (Bio-Rad) for ATXN3 protein analysis. Subsequently, the proteins were wet transferred onto a nitrocellulose membrane (AmershamProtran ) for 1.5 h. Primary and secondary antibodies (Table S4) were dissolved in PBS with 0.1% Tween 20 buffer supplemented with 5% non-fat milk. The membrane was incubated with the primary antibody (anti-HTT, Sigma-Aldrich MAB2166; anti-ATXN3, Proteintech 13505-1-AP; anti-vinculin, Proteintech 26520-1-AP; anti-lamin B1, Proteintech 12987-1-AP) at 4°C overnight and with the secondary antibody (Peroxidase AffiniPure Donkey Anti-Mouse, Jackson ImmunoResearch, 715-035-150, and Peroxidase AffiniPure Donkey Anti-Rabbit, Jackson ImmunoResearch 711-035-152) for 1.5 h at room temperature. Detection was performed using a substrate for horseradish peroxidase (SuperSignal West Pico PLUS Chemiluminescent Substrate, Thermo Scientific). The signal was captured using a G:box system.

### Immunohistochemistry and quantification

The tissues were fixed overnight in ice-cold 4% paraformaldehyde and then cryopreserved in sucrose (22%) for 72 h at 4°C or frozen in liquid nitrogen and stored at −80°C. Brains and livers were embedded in OCT tissue-freezing medium (Leica Microsystems) and frozen on dry ice. The organs were cut using a cryostat at −20°, and 20-μm sagittal sections were collected on SuperFrost Plus slides (Thermo Fisher Scientific). Sections were kept at −80°C and air-dried for 10 min before immunostaining. The sections were processed by incubation in citrate buffer (pH 9.0) for 15 min at 90°C and incubated for 10 min at −20°C. The sections were blocked via incubation in 4% normal goat serum in PBS for 1 h. Primary antibodies anti-HTT and anti-ATXN3 (1:100 MAB5374 clone mEM48; 1:500 MAB5360 clone 1H9 [all from Millipore]) and secondary antibodies conjugated with Alexa Fluor 647 1:500 (Jackson ImmunoResearch, 2340770) were used for immunofluorescence analysis of brain sections (Table S4). Next, brains and liver sections were counterstained with Hoechst 33342 (Sigma-Aldrich; 1:10,000) and coverslipped with Fluoroshield (Sigma-Aldrich). Spinning disc microscopic images were acquired using the microscope system Opera LX (PerkinElmer) and with the same image parameters (brightness-contrast-intensity). Tile images from Opera LX were assembled using ImageJ software (National Institutes of Health) to produce full images of the EGFP^+^ brain sections. The ROI objects covering the individual brain regions (Figure S2) were applied to the green EGFP channel of the full images of the brain sections. Subsequently, the total signal intensity from all pixels (RawIntDen) inside the ROI and the surface area value for ROI were acquired by ImageJ. The relative transduction level of a brain region was expressed as a fluorescent intensity per area unit quantified by normalizing the RawIntDen for the square micrometer ROI area.

Images of brain sections double labeled for HTT or ATXN3 and EGFP were acquired to quantify aggregates. The images were then segmented for areas positive for EGFP labeling. The EGFP^+^ ROIs were then used to quantify aggregates, limiting the quantification to inside transduced cells. In these transduced areas, the aggregate surface was estimated as a percentage of the area covered by aggregates (% EGFP^+^ area), and the intrinsic density of the aggregates was estimated as mean integrated density (mean IntDen).

Single-cell transduction variability was estimated in the Purkinje cell by segmenting cell bodies from lobule 5, measuring the RawIntDen per square micrometer and performing basic descriptive statistics. The data show minima, maxima, median, and standard deviations similar among the datasets from 3 cerebella (Table S5).

### Statistical analysis

The obtained results were analyzed using GraphPad Prism software (version 8.0.1). Statistical tests are specified in the figure legends for each experiment. The protein data are presented as mean or fold change calculated from mean of protein level. Error bars represent the standard error of the mean (±SEM). In the case of the aggregate, the data were analysed with the Mann Whitney test. The data of mRNA were analysed with Kruskal-Wallis test for multiple comparisons and subsequently post hoc analysed with Dunn test. The p values in tests were indicated as follows ∗p < 0.05; ∗∗p < 0.01; ∗∗∗p < 0.001; ∗∗∗∗p < 0.0001.

## Data availability

The additional data and research materials are available on the Zenodo portal repository under https://doi.org/10.5281/zenodo.14984506.

## Acknowledgments

The authors would like to acknowledge the late Włodzimierz J. Krzyżosiak as an initial participant in the general concept of the study. This work was supported by a grant awarded to M.F., Y.T., H.P.N., and M.R.H. from the European Research Projects on Rare Diseases (JTC 2017) (grant no. ERA-NET-E-RARE-3/III/TreatPolyQ/08/2018), provided by the 10.13039/501100005632Polish National Center for Research and Development (to M.F.) by the 10.13039/501100001665Agence Nationale de la Recherche France (ANR-17-RAR3-0008-04_ACT) (to Y.T.). M.F. and Y.T. sincerely thank the 10.13039/100002243National Ataxia Foundation and their donors for their funding support by awarding the Pioneer SCA3/MJD Translational Research Grant (no. 688790). We thank Pascale Kobel for producing the AAV_PhP.eB virus. We thank Dr. Hab. Jacek Kolanowski and Dr. Dorota Kwiatek for sharing their valuable expertise and granting access to the infrastructure of the Centre for Chemical Biology at Institute of Bioorganic Chemistry, Polish Academy of Sciences, Poznań, Poland and acknowledging the associated funding of Project of the Polish Platform of Screening Infrastructure for Biological Chemistry (POL-OPENSCREEN), which was funded by a grant from the 10.13039/501100004569Ministry of Science and Higher Education (decision DIR/WK/2018/06), part of the European Infrastructure of Open Screening Platforms for Chemical Biology European Research Infrastructure Consortium (EU-OPENSCREEN). We thank Adam Plewinski and Dorota Wronka for their expertise and help in maintaining the Hu^128Q/21Q^ and Ki^150Q/21Q^, HD, and SCA3 mouse model colonies.

## Author contributions

Conceptualization: M.S., A.F., H.P.N., Y.T., and M.F. Investigation: M.S., Ż.K.-P., E.J., A.N.-C., M.F., L.F., N.S.C., E.S.-M., M.R.H., and Y.T. Formal analysis, data curation, and visualization: M.S., Ż.K.-P., A.F., L.P., A.N.-C., Y.T., and M.F. Writing – original draft: M.S. and M.F. Funding acquisition and resources: H.P.N., Y.T., M.R.H., and M.F. Supervision: M.F. All authors approved the submitted version of the article.

## Declaration of interests

A.F. is listed as a co-inventor on patents (US9970004B2 and US10329566B2) concerning the application of the RNAi approach in the treatment of diseases caused by the expansion of CAG trinucleotide repeats.

## Supplemental information

Document S1. Figures S1–S3 and Tables S1–S5

Document S2. Article plus supplemental information
