# Supplementary material for: CAG-targeted brain-permeable therapy tested in biallelic humanized polyQ mouse models
Source: Mol Ther Nucleic Acids. 2025 Feb 22;36(2):102496. doi: 10.1016/j.omtn.2025.102496 (PMC11960632; doi:10.1016/j.omtn.2025.102496)
Supplement: Document S1. Figures S1–S3 and Tables S1–S5 [file mmc1.pdf]

**OMTN, Volume *36***

## Supplemental information

# CAG-targeted brain-permeable therapy tested in biallelic humanized polyQ mouse models

**Magdalena Surdyka, Żaneta Kalinowska-Pośka, Anna Niewiadomska-Cimicka, Ewelina Jesion, Agnieszka Fiszer, Elisabeth Singer-Mikosch, Lorraine Fievet, Lukasz Przybyl, Nicholas S. Caron, Michael R. Hayden, Huu Phuc Nguyen, Yvon Trottier, and Maciej Figiel**

**Table S1. Off-target shRNAs in *Homo Sapiens (A)* and *Mus Musculus (B).*** The table summarizes an extensive in silico analysis of the designed CAG-targeting reagents off-targets using the BLAST. To simplify the presentation of the quality of off-targets of oligonucleotide binding sites to mRNA, we applied arbitrary rankings using the bit-score parameter generated by BLAST combined with listing how many nucleotides from a oligonucleotide bind to the mRNA off-target gene. We added color coding and referred arbitrarily to RED as strong off-targets, YELLOW as moderate off-targets, and GREEN as weak off-targets. We are fully aware of the simplistic nature of the presentation of our analysis. However, it allows readers to assess off-targets quickly and avoids presenting extensive Excel tables with complicated analysis parameters. The detailed tables listing all BLAST parameters of alignments are available upon request at mfigiel@ibch.poznan.pl

# (A)

## Off-targets Homo Sapiens

range between 21nt and 18nt; cut-off: 17 and less

### A2

| Gene | bit-score | out of 21: |
|---|---|---|
| CCDC177 | 42,1223 | 21 |
| MINK1 | 42,1223 | 21 |
| MYT1 | 42,1223 | 21 |
| PEG3 | 42,1223 | 21 |
| SLC16A2 | 42,1223 | 21 |
| BRD4 | 40,14 | 20 |
| BSN | 40,14 | 20 |
| SOGA3 | 40,14 | 20 |
| TOX3 | 40,14 | 20 |
| SLFNL1 | 38,1576 | 19 |

### A2+(P10A)

| GENE | bit-score | out of 21: |
|---|---|---|
| CCDC177 | 34,1929 | 20 |
| CIZ1 | 34,1929 | 20 |
| DACH1 | 34,1929 | 20 |
| EDC4 | 34,1929 | 20 |
| KBTBD6 | 34,1929 | 20 |
| KDM6B | 34,1929 | 20 |
| MINK1 | 34,1929 | 20 |
| MLLT3 | 34,1929 | 20 |
| MYT1 | 34,1929 | 20 |
| PEG3 | 34,1929 | 20 |
| RABGAP1L | 34,1929 | 20 |
| RBFOX1 | 34,1929 | 20 |
| SLC16A2 | 34,1929 | 20 |
| TSHZ3 | 34,1929 | 20 |
| ARID1B | 26,2635 | 19 |

### A4

| GENE | bit-score | out of 21: |
|---|---|---|
| ELN | 40,14 | 20 |
| KMT2D | 40,14 | 20 |
| MLLT6 | 40,14 | 20 |
| CCDC177 | 34,1929 | 20 |
| ISL2 | 34,1929 | 20 |
| MINK1 | 34,1929 | 20 |
| MYT1 | 34,1929 | 20 |
| PEG3 | 34,1929 | 20 |
| PIP4P2 | 34,1929 | 20 |
| RIMBP3 | 34,1929 | 20 |
| SLC16A2 | 34,1929 | 20 |
| TFAP4 | 34,1929 | 20 |
| TRERF1 | 34,1929 | 20 |
| KMT2D | 30,2282 | 19 |

### A4+(P10A)

| GENE | bit-score | out of 21: |
|---|---|---|
| ELN | 32,2105 | 19 |
| KMT2D | 32,2105 | 19 |
| LINC01209 | 32,2105 | 19 |
| MLLT6 | 32,2105 | 19 |
| WDR97 | 32,2105 | 19 |
| BCAR1 | 30,2282 | 18 |
| CHD8 | 30,2282 | 18 |
| RIMBP2 | 30,2282 | 18 |

### A4+(P10,11A)

| GENE | bit-score | out of 21: |
|---|---|---|
| PPP2R5E | 34,1929 | 17* |

*No off-targets with cut-off values 21-18 nucleotides were found

### AA4

| GENE | bit-score | out of 21: |
|---|---|---|
| ACSS1 | 24,2811 | 18 |

### AG4

| GENE | bit-score | out of 21: |
|---|---|---|
| CCDC177 | 34,1929 | 20 |
| MINK1 | 34,1929 | 20 |
| MYT1 | 34,1929 | 20 |
| PEG3 | 34,1929 | 20 |
| RIMBP3 | 34,1929 | 20 |
| SLC16A2 | 34,1929 | 20 |
| TNRC6A | 34,1929 | 20 |
| CCAR1 | 36,1753 | 18 |

### A15

| GENE | bit-score | out of 21: |
|---|---|---|
| PPARD | 30,2282 | 15* |

*No off-targets with cut-off values of 21-16 nucleotides were found

**(B)**

# Off-targets Mus Musculus

range between 21nt and 18nt; cut-off: 17 and less

## A2

| GENE | bit-score | out of 21: |
|---|---|---|
| Ccdc177 | 42,1223 | 21 |
| Maml1 | 42,1223 | 21 |
| Ppp1r3f | 42,1223 | 21 |
| Golga4 | 40,14 | 20 |
| Soga3 | 40,14 | 20 |
| Th | 40,14 | 20 |
| Mink1 | 38,1576 | 20 |

## A2+(P10,11A)

| GENE | bit-score | out of 21: |
|---|---|---|
| Colec11 | 34,1929 | 20 |
| Gm41539 | 34,1929 | 20 |
| Dock9 | 32,2105 | 19 |
| Kmt2d | 30,2282 | 19 |
| Tox3 | 32,2105 | 19 |
| Srrm2 | 30,2282 | 18 |

## A4

| GENE | bit-score | out of 21: |
|---|---|---|
| Stc1 | 42,1223 | 21 |
| A230006K03Rik | 40,14 | 20 |
| Pum1 | 40,14 | 20 |
| D6Ertd527e | 34,1929 | 20 |
| Mad1l1 | 34,1929 | 20 |
| Tbl1x | 34,1929 | 20 |
| Zfp853 | 34,1929 | 20 |
| Bsn | 36,1753 | 18 |
| Zfp804a | 36,1753 | 18 |

## A4+(P10A)

| GENE | bit-score | out of 21: |
|---|---|---|
| Gm41539 | 34,1929 | 20 |
| Kmt2d | 34,1929 | 20 |
| Stc1 | 34,1929 | 20 |
| A230006K03Rik | 32,2105 | 19 |
| Pum1 | 32,2105 | 19 |
| Tox3 | 32,2105 | 19 |

## A4+(P10,11A)

| GENE | bit-score | out of 21: |
|---|---|---|
| Kmt2d | 34,1929 | 20 |
| Gm16069 | 32,2105 | 19 |

## AA4

| GENE | bit-score | out of 21: |
|---|---|---|
| Kmt2d | 26,2635 | 19 |
| LOC115489118 | 26,2635 | 19 |

## AG4

| GENE | bit-score | out of 21 |
|---|---|---|
| Ccdc177 | 34,1929 | 20 |
| Lrch3 | 34,1929 | 20 |
| Maml1 | 34,1929 | 20 |
| Ppp1r3f | 34,1929 | 20 |
| Stc1 | 34,1929 | 20 |
| A230006K03Rik | 32,2105 | 19 |
| Golga4 | 32,2105 | 19 |
| Zfhx3 | 26,2635 | 19 |

## A15

| GENE | bit-score | out of 21 |
|---|---|---|
| LOC105245978 | 28,2458 | 17* |

*No off-targets with cut-off values of 21-18 nucleotides were found

**Table S2. Titer and dose of reagents used for retro-injection.**

| Name | Titer [vg/ul] | Dose [vg/kg] |
|---|---|---|
| Scrambled | $2.20x10^{10}$ | $1.5 \times 10^{13}$ |
| A15 | $4.30x10^{10}$ | $1.5 \times 10^{13}$ |
| A4 | $3.10x10^{10}$ | $1.5 \times 10^{13}$ |
| A2 | $5.00x10^{9}$ | $0.5 \times 10^{13}$ |
| A2(P10,11A) | $9.80x10^{9}$ | $1.5 \times 10^{13}$ |
| AG4 | $1.30x10^{10}$ | $1.5 \times 10^{13}$ |
| A4(P10A) | $1.90x10^{10}$ | $1.5 \times 10^{13}$ |
| A4(P10A,11A) | $2.10x10^{10}$ | $1.5 \times 10^{13}$ |
| AA4. | $2.50x10^{10}$ | $1.5 \times 10^{13}$ |

**Table S3.** Sequence of primers used for RT-qPCR

| Target gene | Primer orientation | Sequence |
|---|---|---|
| *HTT* | F | GAGCCGCTGCACCGAC |
| | R | CTGACAGACTGTGCCACTATGTTT |
| *ATXN3* | R | CGCAGGGCTATTCAGCTAAG |
| | R | GCTTTTGCTGCTGTTTTTCA |
| *Actin* | F | TTCTTTGCAGCTCCTTCGTT |
| | R | ATGGAGGGGAATACAGCCC |

**Table S4. Antibodies used for Western blot analysis and immunohistochemistry**

| Antibody | Type | Host | Dilution | Factory |
|---|---|---|---|---|
| ***Primary antibody*** | | | | |
| Huntingtin | monoclonal | mouse | 1:1000 (WB) | Sigma-Aldrich MAB2166 |
| ATXN3 | polyclonal | rabbit | 1:1000 (WB) | Proteintech 13505-1-AP |
| Vinculin | polyclonal | rabbit | 1:5000 (WB) | Proteintech 26520-1-AP |
| Lamin B1 | polyclonal | rabbit | 1:5000 (WB) | Proteintech 12987-1-AP |
| Anti-Huntingtin | monoclonal | mouse | 1:100 (IHC) | Sigma-Aldrich MAB5374 |
| ATXN3 | monoclonal | mouse | 1:500 (IHC) | Sigma-Aldrich MAB5360 |
| Hoechst33342 | | | 1:5000 (IHC) | Sigma Aldrich |
| ***Secondary antibody*** | | | | |
| Peroxidase AffiniPure Donkey Anti-Mouse | polyclonal | donkey | 1:5000 (WB) | Jackson ImmunoResearch 715-035-150 |
| Peroxidase AffiniPure Donkey Anti-Rabbit | polyclonal | donkey | 1:5000 (WB) | Jackson ImmunoResearch 711-035-152 |
| AlexaFluor647 | polyclonal | goat | 1:500 (IHC) | Jackson ImmunoResearch 2340770 |

**Table S5. Single-cell transduction variability.** The variability was estimated in the Purkinje cell by segmenting cell bodies from lobule 5 of 3 cerebella, measuring the RawIntDen per um2, and performing basic descriptive statistics. The data show minima, maxima, median, and standard deviations similar among the datasets from 3 cerebella. The table is also available as a separate Excel file in supplementary materials.

| # | Cerebellum 1 | Cerebellum 2 | Cerebellum 3 |
|---|---|---|---|
| 1 | 10179,67306 | 11158,3777 | 8518,857979 |
| 2 | 10782,79578 | 11118,13724 | 6209,466951 |
| 3 | 11212,46566 | 9568,819243 | 4975,868341 |
| 4 | 9098,269053 | 10834,77929 | 9692,198829 |
| 5 | 4879,625622 | 10871,32047 | 10418,07089 |
| 6 | 9873,716408 | 11212,46566 | 10260,93986 |
| 7 | 10415,0558 | 11034,56412 | 10055,79111 |
| 8 | 3504,189757 | 10074,87557 | 11280,97706 |
| 9 | 9245,637129 | 4973,319213 | 10236,34119 |
| 10 | 11246,39669 | 10349,05834 | 6709,341723 |
| 11 | 6848,072345 | 10836,62987 | 9742,599598 |
| 12 | 9684,78625 | 10183,32527 | 11452,89475 |
| 13 | 6190,770105 | 7291,767506 | 10634,4901 |
| 14 | 6271,243562 | 9944,417506 | 11274,91666 |
| 15 | 8872,650208 | 9792,720122 | 11411,89182 |
| 16 | 6119,309151 | 6737,274121 | 11307,93081 |
| 17 | 6760,578254 | 7173,650863 | 7325,613575 |
| 18 | 9275,760737 | 7621,574938 | 11193,38723 |
| 19 | 7061,153594 | 6012,780045 | 11206,30983 |
| 20 | 11243,07897 | 7185,156435 | 3840,26655 |
| 21 | 8022,479253 | 6389,280438 | 10975,34766 |
| 22 | 6351,837155 | 11007,03518 | 7703,117234 |
| 23 | 7621,867269 | 7193,681021 | 9992,445884 |
| 24 | 6489,352508 | 10604,4755 | 8336,504488 |
| 25 | 9274,585943 | 6351,837155 | 8559,989762 |
| 26 | 5162,022805 | 7002,108379 | 2570,958555 |
| 27 | 10987,33274 | 6295,174839 | 7702,242281 |
| 28 | 11289,71646 | 9626,613967 | 10553,97397 |
| 29 | 3780,411735 | 5194,461295 | 9320,69216 |
| 30 | 3356,64941 | 11215,68769 | 6417,833303 |
| 31 | 2737,925747 | 11382,55827 | 7918,316049 |
| 32 | 5462,837757 | 3482,807249 | |
| 33 | 3913,62745 | 2692,548484 | |
| 34 | 10049,30034 | 5462,837757 | |
| 35 | 2223,445163 | 9459,359074 | |

| Number of values | 43 | 42 | 31 |
|---|---|---|---|
| | | | |
| Minimum | 2223 | 2134 | 2571 |
| 25% Percentile | 5620 | 6380 | 7702 |
| Median | 8022 | 9598 | 9743 |
| 75% Percentile | 10186 | 10841 | 10975 |
| Maximum | 11290 | 11383 | 11453 |
| Range | 9066 | 9248 | 8882 |
| | | | |
| 95% CI of median | | | |
| Actual confidence level | 96,85% | 95,64% | 97,06% |
| Lower confidence limit | 6352 | 7174 | 7918 |
| Upper confidence limit | 9685 | 10183 | 10554 |
| | | | |
| Mean | 7771 | 8419 | 8961 |
| Std. Deviation | 2707 | 2597 | 2356 |
| Std. Error of Mean | 412,8 | 400,8 | 423,2 |

| | | | |
|---|---|---|---|
| 36 | 5620,037836 | 5681,525462 | |
| 37 | 7009,733503 | 2134,302678 | |
| 38 | 10559,98119 | 7009,733503 | |
| 39 | 8450,416143 | 11089,81915 | |
| 40 | 10186,40151 | 9699,960127 | |
| 41 | 10977,0463 | 9780,750386 | |
| 42 | 5099,88038 | 10852,68655 | |
| 43 | 10756,06705 | | |

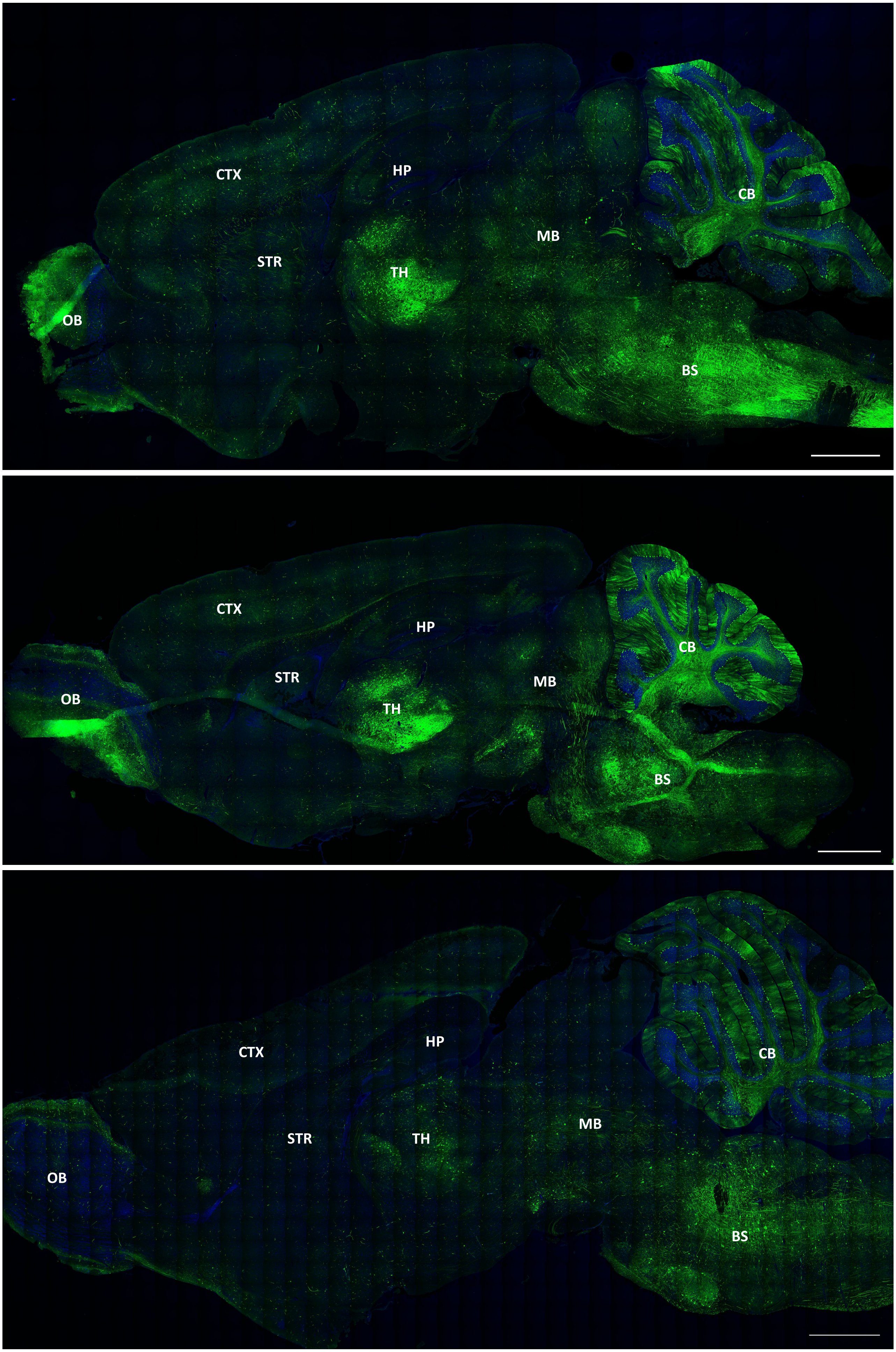


**Figure S1**. The 3 brain images (3 weeks post-injection) were assembled in Fiji ImageJ from tile images from Opera Phenix, and used for further processing and ROIs.

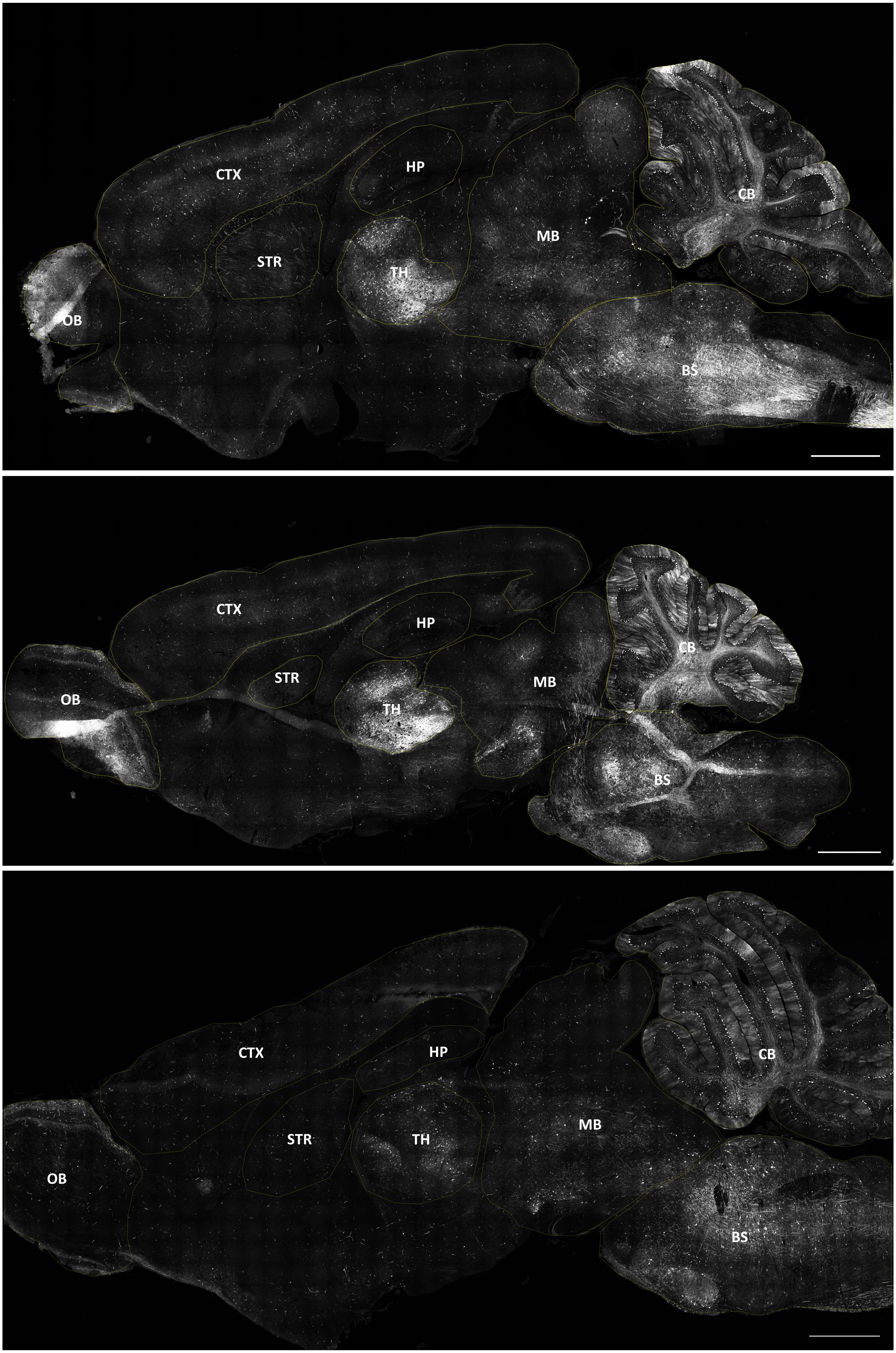


**Figure S2**. ROIs used for segmenting brain regions of 3 brains **(Figure S1)**, used for acquiring fluorescence intensity by RawIntDen of eGFP signal on a single channel in Fiji ImageJ (**Figure 1B**).

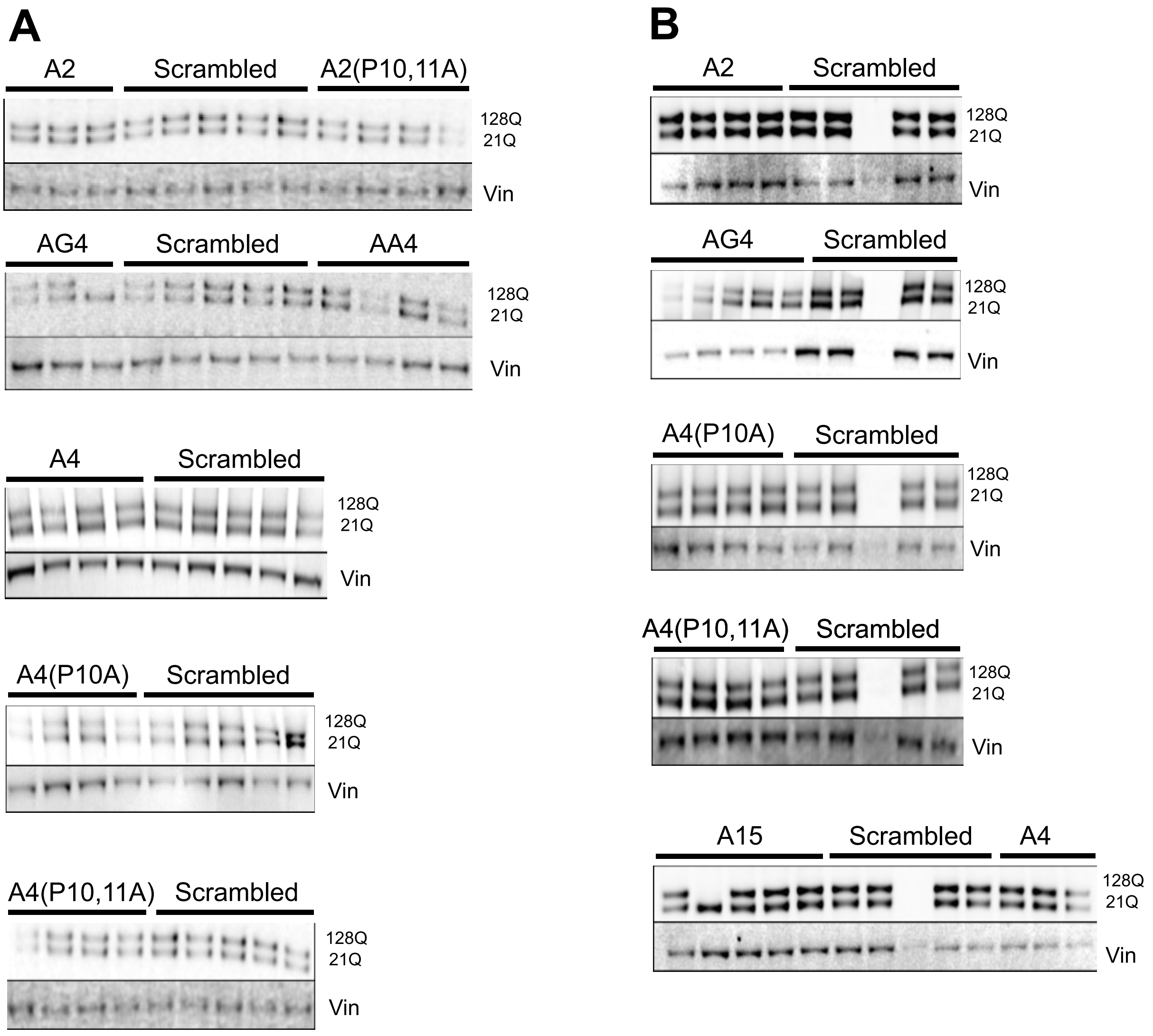


**Figure S3**. Western blotting membranes of the initial evaluation of the efficiency of 8 shRNA reagents in lowering HTT protein in the thalamus (**Figure S1A**), and striatum (**Figure S1B**) of 3 weeks post-injection (**Figure 1C**).
